# Patient‐Reported Outcomes and Quality of Life Assessment in Breast Surgery Using BREAST‐Q: A Multi‐Center Prospective Study

**DOI:** 10.1002/wjs.70131

**Published:** 2025-10-07

**Authors:** Megan Roy Pickard, Sarkis Meterissian

**Affiliations:** ^1^ Faculty of Medicine and Health Sciences McGill University Montreal Quebec Canada; ^2^ Department of Surgery McGill University Montreal Quebec Canada

The increasing longevity of breast cancer survivors worldwide has shifted surgical priorities beyond oncologic safety toward optimizing quality of life. Patient‐reported outcomes (PROs) have become central to modern surgical decision‐making, recognizing that survival alone is an insufficient measure of success. In this context, Selvarajoo et al. present a prospective, multi‐center Malaysian study comparing breast‐conserving surgery (BCS) to mastectomy using the BREAST‐Q [1]. The study enrolled 230 women with stage 0–III disease (50.4% BCS, 49.6% mastectomy) and achieved over 90% follow‐up at 12 months, with validated local‐language questionnaires administered preoperatively and at 6 and 12 months [1].

Across all time points, BCS patients reported significantly higher breast satisfaction and superior psychosocial and sexual well‐being compared to mastectomy [1]. At 12 months, physical well‐being outcomes also favored BCS [1]. These differences align with evidence from long‐term comparative studies and are likely driven by preservation of breast contour and sensation, promoting better body image and sexual well‐being [2]. Interestingly, in this study, none of the mastectomy patients received reconstruction, whether by study design, patient preference or due to limited resources [1]. The absence of reconstruction strengthens the global relevance of the findings by offering a clean comparison of two primary surgical options, but it also raises the question of how results might differ if reconstruction were available. Evidence from other settings suggests that implant‐based reconstruction after mastectomy can narrow, but not eliminate, the advantages of BCS [2]. Only immediate autologous reconstruction achieves comparable or superior scores in these domains [3]. However, autologous procedures are substantially more complex, requiring microsurgical expertise, prolonged operative time, greater resource allocation, and higher overall costs, all factors that limit feasibility in many health systems [3]. This context underscores that the observed BREAST‐Q gap is not solely a function of reconstruction access, and that even when reconstruction is offered, BCS often retains a quality‐of‐life advantage [2, 3].

The findings are particularly salient for low‐ and middle‐income countries (LMICs), where mastectomy without reconstruction remains the default surgical approach, not because it optimizes quality of life, but because it is reliably deliverable [4]. Limited access to adjuvant radiotherapy, critical for achieving local control, often drives clinicians to recommend mastectomy, even for patients who could otherwise be candidates for breast conservation. The higher proportion of later‐stage diagnoses, frequently with larger tumors, further reduces the pool of women eligible for BCS [5]. In some contexts, the surgical workforce is composed largely of general surgeons without subspecialty training in breast oncology, making mastectomy a technically simpler and faster option. Fear of recurrence, shared by both patients and providers, further reinforces this tendency despite decades of evidence demonstrating no difference in survival or local recurrence between BCS with radiation and mastectomy [4]. Where oncologic outcomes are equivalent, increasing BCS availability is a straightforward way to improve postoperative quality of life without affecting survival [5]. International guidelines emphasize timely access to surgery and earlier diagnosis, with the goal of detecting at least 60% of breast cancers at stage I–II, thereby increasing eligibility for BCS [5]. Incorporating PROs such as the BREAST‐Q into routine planning can help ensure surgical decisions address both tumor removal and long‐term patient outcomes.

Notably, no BCS patients in the cohort required re‐excision for positive margins, an outcome uncommon even in high‐resource settings, and one that likely contributed to more favorable PROs by sparing patients the physical, psychological, and logistical burdens of additional surgery [1]. Paradoxically, mastectomy patients reported greater satisfaction with their surgeon and medical team despite lower overall well‐being scores [1]. Differences in satisfaction may be related to the older age profile of the mastectomy group or perhaps the perception that more extensive procedures require greater technical skill. However, the absence of multivariable analysis leaves room for residual confounding. In particular, the lack of stratification by age is important, as older age can influence both surgical choice and satisfaction. Older women may be more inclined to choose mastectomy and more likely to value physician‐led decision‐making, which could affect PROs independently of surgical approach. Without adjustment for factors such as age, tumor stage, adjuvant therapy, socioeconomic status, and comorbidities, it is difficult to determine the extent to which the observed differences in PROs are attributable to the surgery itself rather than study group characteristics.

Importantly, BCS itself has evolved, with oncoplastic breast‐conserving surgery (OBCS) offering further quality‐of‐life gains [5]. By integrating wide local excision with immediate reshaping, OBCS extends eligibility to women with larger tumors or less favorable breast morphology who might otherwise require mastectomy [5]. Evidence has shown that OBCS can match or surpass traditional BCS in breast satisfaction and psychosocial well‐being, while maintaining acceptable complication rates [5]. Beyond esthetic benefits, volume‐displacement and reduction techniques can optimize radiotherapy delivery and reduce post‐treatment asymmetry [5]. Expanding surgeon training in oncoplastic techniques could ensure that, once BCS capacity is secured, patients globally have access to the most advanced approaches for achieving both oncologic safety and long‐term quality of life.

To conclude, when survival outcomes are equivalent, the priority should be to choose a surgical approach that maximizes quality of life. Preserving the breast when oncologically safe should be the standard of care, supported by adequate surgical training, access to radiotherapy, and routine integration of PRO measurement into decision‐making. In this light, the authors' direct comparison of BCS and mastectomy without reconstruction provides particularly valuable evidence with global applicability [1]. By delivering high follow‐up rates, using rigorously validated local‐language instruments, and representing a broad spectrum of stage 0–III disease, this work offers robust, context‐specific insights that are often lacking in the literature. For patients and health systems alike, the challenge is not simply to save lives, but to ensure those lives are lived well, and this study makes a meaningful contribution toward that goal.

## Author Contributions

The authors contributed equally to this manuscript.

## Conflicts of Interest

The authors declare no conflicts of interest.
